# ARCADO - Adding random case analysis to direct observation in workplace-based formative assessment of general practice registrars

**DOI:** 10.1186/s12909-015-0503-2

**Published:** 2015-12-10

**Authors:** Gerard Ingham, Jennifer Fry, Simon Morgan, Bernadette Ward

**Affiliations:** Beyond Medical Education, PO Box 3064, Bendigo, Victoria 3550 Australia; GP Training Valley to Coast, Hunter Regional Mail Centre, PO Box 573, Newcastle, NSW 2310 Australia; School of Rural Health, Monash University, PO Box 666, Bendigo, 3550 Victoria Australia

**Keywords:** Workplace-based assessment, Formative assessment, General practice training, Clinical supervision

## Abstract

**Background:**

Workplace-based formative assessments using consultation observation are currently conducted during the Australian general practice training program. Assessment reliability is improved by using multiple assessment methods. The aim of this study was to explore experiences of general practice medical educator assessors and registrars (trainees) when adding random case analysis to direct observation (ARCADO) during formative workplace-based assessments.

**Methods:**

A sample of general practice medical educators and matched registrars were recruited. Following the ARCADO workplace assessment, semi-structured qualitative interviews were conducted. The data was analysed thematically.

**Results:**

Ten registrars and eight medical educators participated. Four major themes emerged - formative versus summative assessment; strengths (acceptability, flexibility, time efficiency, complementarity and authenticity); weaknesses (reduced observation and integrity risks); and contextual factors (variation in assessment content, assessment timing, registrar-medical educator relationship, medical educator’s approach and registrar ability).

**Conclusion:**

ARCADO is a well-accepted workplace-based formative assessment perceived by registrars and assessors to be valid and flexible. The use of ARCADO enabled complementary insights that would not have been achieved with direct observation alone. Whilst there are some contextual factors to be considered in its implementation, ARCADO appears to have utility as formative assessment and, subject to further evaluation, high-stakes assessment.

## Background

Over the last 40 years there has been a shift to competency-based medical education [1]. This has been accompanied by new assessment principles and the increasing use of formative assessment and workplace-based assessment [2].

Prime amongst the new assessment principles is to measure what is important rather than just what can be measured [3]. A master plan, or blueprint, should be used that matches assessments against desired outcomes [4]. This purposeful approach improves the overall validity of the assessment program [5]. Overall reliability is increased by using multiple assessment methods [6].

Formative assessment occurs during training, and is low-stakes assessment *for* learning. It differs from summative assessment which is high stakes assessment *of* learning that typically occurs at the end of training. Formative assessment aims to generate powerful learning experiences from feedback [7]. Careful selection of the formative assessment methods should steer learning towards desired outcomes [8].

Workplace-based assessments can assess what a person actually does (performance) rather than just what a person is capable of doing (competence). Ultimately it is more important to know what is being done as a result of learning rather than just what can be done [9].

Despite the emphasis placed on workplace-based formative assessments during training, there are few published articles on their impact on doctor’s education and performance [10]. Further research has been recommended including investigating the use of combined workplace-based formative assessments [11].

Two assessment methods currently used in workplace-based formative assessment are observation of consultations and review of clinical records. Consultations can be observed by reviewing video recordings or by direct observation (DO). Clinical records can be reviewed in a number of ways including random case analysis (RCA) where the assessor selects the record and discusses the case with the learner [12].

DO is used to assess a wide variety of competencies, with clinical skills, communication skills, and professionalism being the most readily assessed [13]. RCA similarly has the capacity to assess broadly but has particular utility in assessing clinical knowledge, clinical reasoning, and the quality of medical record keeping [14, 15]. RCA has been found to have particular value in detecting registrar ‘unknown unknowns’ and patient safety concerns [16] although compared to DO its role in high-stakes assessment is less well established [17].

A common setting for workplace-based formative assessments in the Australian general practice (GP) training program is the external clinical teaching visit (ECTV) [18]. The ECTV is a visit by a trained GP medical educator to the workplace of the GP registrar (trainee). Direct observation of consultations has long been the mainstay of assessment in the ECTV [19].

In keeping with current emphasis on the use of multi-modal or combined workplace-based formative assessments, we proposed the addition of RCA to DO of consultations in the ECTV. The combination of assessments, named ARCADO, has face validity as a formative assessment, but it was unknown how this would work in practice. The aim of this study was to explore the experiences and perceptions of medical educators and GP registrars of the addition of RCA to DO within the ECTV as a formative assessment. Specifically, we asked whether ARCADO was acceptable to educators and registrars and if it was useful in driving learning. We also sought whether registrars and educators perceived that ARCADO is a valid assessment method, and whether there are there contextual factors that influence the validity, acceptability and usefulness in driving learning.

## Methods

A qualitative research design was used. Qualitative research aims to describe participant’s perceptions or experiences and is useful when little is known about a phenomenon [20].

### Setting and recruitment

The setting was an Australian Regional Training Provider (RTP) of postgraduate vocational general practice training. In November 2014, all medical educators from one region of the RTP who attended a two-day professional development workshop that included a two-hour training session in the use of the ARCADO assessment tool were invited to be included in the study. Excluded medical educators were those assessing registrars in remediation and those who were in the expert reference group. Eligible medical educators and their matched registrars were subsequently emailed by RTP administrative staff seeking consent for the researcher (JF) to make contact about the study. Invitees were emailed information about the use of DO and RCA in ECTVs and study explanatory statements. Written informed consent for participation in the study was obtained from all medical educators and registrars.

### Assessment tool

The ARCADO assessment tool was adapted from an existing tool used by the RTP for consultation observation assessment in the ECTV. This tool consisted of three sections. The first section, containing a brief overview of each clinical case, was adapted to include whether the case was DO of a consultation or RCA of a medical record. The second section prompted feedback about the registrar’s skills in history taking, examination, clinical reasoning and problem definition, management, explanation and planning, context of the consultation, professional, ethical and legal, organisational and medical records. The final section asked for an overall assessment of the registrar’s competence for the current stage of training and a review of the registrar’s learning plan in the context of the assessment. There were also prompts to discuss with the registrar what he/she should keep doing, do more of, do less of, and stop doing.

### Data collection

Individual semi-structured interviews were conducted with the medical educators and the registrars by JF within 7 days of the ECTV. Data was collected face-to-face, via video-conferencing or the telephone. Interviews were audio-recorded and were 45–60 minutes long. Recruitment continued until data saturation was reached [21].

### Data collection tools

The qualitative interview was based on medical pedagogical literature [6, 8] and input from an expert reference group. The semi-structured questions explored the context of the ECTV, perceptions and acceptability of DO and RCA, confidence in the assessment of overall competence, and usefulness in identifying learning needs.

Data collected about the participants and the assessments included socio-demographics, medical educator and GP registrar education and experience, and contextual factors about the ECTV (number of patients seen, records reviewed and assessment outcome).

The qualitative interview guide was pre-tested with a medical educator and registrar and this resulted in minor changes to the questions. The interview guide is available on request.

### Analysis

The qualitative interviews were transcribed verbatim and coded using thematic analysis in NVivo [22]. All researchers read transcripts progressively through the data collection stage and met six times to critically reflect on the transcripts and to determine when data saturation was reached. Two researchers (JF and BW) independently read, re-read and coded the transcripts and met to conduct an inter-rater reliability assessment. Greater than 80 % agreement was reached for all items [23]. In the second stage of analysis, the four researchers met to reach agreement on the identification of themes and grouping like codes together [21].

### Trustworthiness

The study design and methods were informed by an expert reference group that met pre and post data collection. The Consolidated Criteria for Reporting Qualitative Research (COREQ) guidelines were used to guide the reporting of the study [24]. A reflexive approach was adopted with supervision by the non-aligned Monash University employed researcher BW. JF is a non-GP allied health researcher and collected the data.

The study was approved by Monash University Human Research Ethics Committee.

## Results

There were eleven eligible medical educators invited and all agreed to participate in the study, but four corresponding registrars (two for one medical educator) declined leaving eight medical educator participants. Two medical educators were interviewed twice resulting in ten ECTV ARCADO assessments of 10 registrar participants.

The characteristics of the participants are included in Table 1.

**Table 1:** Characteristics of medical educators and registrars

Characteristic	Medical Educator *n* = 8	Registrar *n* = 10
Age		
Less than 30	0	3
30 - 39 years	3	5
40 - 49	2	2
50 - 60	2	0
60 plus years	1	0
Gender		
Male	4	6
Female	4	4
Location of primary medical degree		
Australia	8	6
Other	0	4
Number of years employed in GP medical education		
0 - 5	2	N/A
6 - 10	2	
11 - 20	4	
Current term of GP training		
0 - 6 months		3
7 - 12 months	N/A	4
13 - 18 months		3
19 - 24 months		0

The ECTV visits consisted of between two and five DO consultations and between two and seven RCA review of records. The medical educators reported that all of the registrars were assessed to be at the standard for their level of training. Patient safety issues were reported only infrequently in the assessment and were identified with both RCA and DO.

The themes that emerged from the data are shown in Table 2.

**Table 2:** Themes identified from semi structured interviews with medical educators and registrars

Formative versus summative assessment	
Strengths	
Acceptability	
Flexibility and time efficiency	
Complementarity	
Authenticity	
Weaknesses	
Reduced observation	
Integrity risks	
Context	
Variation in assessment content	
Assessment timing	
Registrar-medical educator relationship	
Medical educator’s approach	
Registrar ability	

### Formative versus summative assessment

The ARCADO assessment tool was used as a learning-oriented formative process rather than as a summative assessment by most educators and registrars. There was a greater focus on identifying registrar learning needs than on assessing current competency.

A few registrars described difficulty in dismissing the idea that there was an element of summative assessment.

*So I was told repeatedly it wasn’t an assessment and the first thing said when he came in was “This is not an assessment it’s a learning tool to help you learn”. Despite all that, it still feels sort of like an assessment.* Registrar 9.

As well, medical educators described adopting a greater summative assessment focus when patient safety issues arose.

### Strengths

#### Acceptability

The addition of RCA to DO was well accepted by all participants. Medical educators and registrars were highly supportive of including RCA as an addition to DO in the ECTV, provided there were still an adequate number of observed consultations to enable assessment of communication and consultation skills.

*I would never want to withdraw clinical observation but I would want to do RCA’s in all future ECTVs.* Medical Educator 6.

RCA was reported by both medical educators and registrars as less stressful for registrars. As a consequence of this observation, there was some discussion that it might be beneficial to commence assessments with RCA rather than with DO.

*RCA was much more comfortable because you don’t have the pressure of making a diagnosis or management plan and you are just looking back on what have you done well, or what you would have done differently.* Registrar 4.

#### Flexibility and time-efficiency

Medical educators appreciated the increased flexibility within the ECTV afforded by the addition of RCA to DO. It provided a ready alternative assessment and learning tool if a patient did not arrive for a consultation. RCA allowed a more time-efficient review of a registrar’s clinical knowledge and reasoning than DO. If the case being reviewed was straightforward it was possible to move on to another.

*It’s quite an efficient way of picking things up. Rather than going through spending 10–20 minutes talking with the patient, with random case analysis you only need to look at it for a couple of minutes and you can identify the needs.* Registrar 6.

There was also, at times, the opportunity for more in depth discussion using RCA because of the absence of the time–pressure created by a waiting patient that may be experienced with DO.

#### Complementarity

Both medical educators and registrars considered that adding RCA to the ECTV improved the comprehensiveness of the assessment by providing a complementary assessment of the consultation. DO tended to focus on communication and examination skills, whereas RCA better assessed clinical knowledge, clinical reasoning and record keeping.

*Direct observation allows you to physically see the interactions and communication. RCA allows you to more quickly go through a number of patients and choose a number of issues.* Medical Educator 5.

*I liked having the random case analysis added in. You have an opportunity to ask questions around the records.* Medical Educator 2.

Some medical educators noted it was possible to reinforce learning issues identified by DO during the subsequent RCA component of the assessment. They described being able to look at the same issue through a different lens.

*I could tell quite early on from observing her consultations that communication skills were very adequate and I found that being able to go to the notes I was able to look at extra things.* Medical Educator 8.

#### Authenticity

Conducting consultations whilst being observed was described as an artificial environment by both medical educators and registrars. The registrar’s behaviour in real life may not match what was being observed.

*So the ECTV is just that session - I can pretend to be a very good doctor and disappear.* Registrar 10.

Adding RCA to the ECTV enabled medical educators to notice differences between observed performance during the visit and actual performance recorded in previous consultations. For example, one medical educator noted records containing many short consultations and a lack of preventative health screening that contrasted with the registrar’s thoroughness during the observed consultations.

Adding RCA enabled an overview of the complexity, scope of practice and types of cases being managed by the registrar. This could raise issues for the medical educator to address with the registrar and practice.

*I’m thinking “well they’re not seeing a great range of patients; we need to be thinking about how that might be improved in the practice itself”. A male registrar might not be getting exposure to female patients or somebody might not be seeing many children, they might be just having an elderly cohort with lots of chronic problems.* Medical Educator 3.

The ability to look back through the notes in RCA enabled educators to uncover how a registrar managed a patient over time rather than just the snap-shot of the consultation observed. This afforded a greater understanding of the registrar’s clinical reasoning and tolerance of uncertainty.

### Weaknesses

#### Reduced observation

Observing and developing communication and consultation skills was considered paramount by medical educators. Some medical educators and registrars were concerned that taking time away from observing consultations during the ECTV decreased the opportunity to assess communication and consultation skills, particularly if less than three patients were observed.

*Three (DOs) might not be enough. If you only had three colds you don’t get to see much.* Medical Educator 1.

#### Integrity risks

Both medical educators and registrars were aware of risks to the integrity of the assessment if the unpredictability of file selection for RCA was not maintained. They also identified the potential for registrars to maintain better records just prior to the ECTV in anticipation that recent records were more likely to be selected for review. Although targeted selection of records had educational advantages this needed to be managed carefully.

*If I put her on the hop, and say, “is there someone you saw yesterday who has a lot of co-morbidities?” we could choose that one as long there is no warning to them that this might happen.* Medical Educator 3.

### Context

#### Variation in assessment content

The advice to medical educators, registrars and practices was to book three DO patient visits and allow 45 minutes for RCA. In practice, the amount of time spent on each component of the assessment and the number of consultations observed and files reviewed varied between the visits.

When patients failed to attend for appointments, the time available for RCA and feedback on DO was longer, but medical educators were concerned that they had inadequate opportunity to assess consultation and communication skills. More frequently, the DO component of the ECTV was longer. In this circumstance, medical educators were mindful of a reduction in the time available for quality feedback on both the RCA and observed consultations. Factors associated with a longer consultation observation time included: patient complexity, registrars being more thorough in response to observation, involvement of other members of the practice team, practices booking more consultations than advised and smaller practices unable to deflect clinical load to other clinicians.

*I took longer than I normally would have done, having an observer, because partly I think I was aware of the observer there and so I paid more attention to the patient and trying to show that I have good communication skills.* Registrar 2.

The value of RCA was reduced when limited notes were recorded or when the notes being reviewed were not recent and the registrar had difficulty recalling the content of the consultation. In these circumstances a few medical educators expressed concern that registrars could possibly embellish their recall.

*Sometimes it’s hard to remember what actually happened when you do the random case analysis from notes. There’s bound to be somethings you can’t remember and this confounds the findings a little bit.* Registrar 2.

#### Assessment timing

ECTVs booked early in the registrar’s practice placement tended to increase the proportion of new patients being seen by the registrar in the assessment. This reduced the capacity of medical educators to assess the registrar’s ability to manage chronic illness and utilise practice and local resources. Some registrars and medical educators reported that the stage of registrar training might also influence the relative value of RCA and DO. They thought DO was of greater importance early in training to focus on communication and consultation skills, but once it was clear that a registrar was competent in these areas, RCA could be used to greater effect.

*My assumption is that I will get better at my consultation skills, so the reasons that you would need to be observed consulting become less and less. I still think there’s a role for it, because there’s always going to be things that might come up that you don’t recognise you’re doing at the time.* Registrar 9.

#### Registrar-medical educator relationship

The relationship between the medical educator and registrar was seen to influence the assessment. Differences were noted in having a younger educator or an educator who was familiar with the registrar or had conducted previous assessments of the registrar. There were diverging views as to whether this had a positive or negative impact. For example, there was some support for medical educators visiting registrars on consecutive ECTVs to enable them to follow the registrar’s progress, but also support for different medical educators to provide varied input into registrars’ learning.

#### Medical educator’s approach

The individual framework of medical educators influenced the assessment. Some medical educators described themselves as being ‘registrar-centred’ and tailoring the ECTV accordingly. Others placed a higher value on assessing particular skills such as consultation skills or the quality of record keeping. Some educators stated a preference for one form of assessment, DO or RCA, over another.

*I like to be able to see the patient and see what’s happening to sort of formulate what I think is going on, so I do find the random case challenging personally.* Medical Educator 1.

#### Registrar ability

Medical educators expressed greater confidence in their ability to assess registrars they perceived to be higher performing. Educators were less certain that the ARCADO ECTV had provided enough information to provide an assessment of competence (summative assessment) when the registrar was considered in the mid to lower range of performance. For the lower performing registrars, medical educators considered a single ARCADO assessment inadequate to determine competence. This contrasted to the use of ARCADO as formative assessment where both RCA and DO were considered valuable in identifying and driving learning for registrars of all abilities.

*Both (DO and RCA) will be advantageous in different ways for the high performing and low performing registrars. I don’t think one or other are geared to either type of registrar.* Medical Educator 5.

## Discussion

We have found that the addition of RCA to DO of consultations is acceptable to both GP registrars and medical educator visitors. The flexibility and time-efficiency of the assessment was regarded as an asset for a formative assessment. The addition of RCA helped overcome some of the artificiality of an assessment based solely on DO. It became more an assessment of ‘does’ than ‘shows how’ [9]. The complementary perspectives of RCA and DO were considered by the participants to enable a more valid assessment.

Although our research considered the use of RCA and DO in a combined assessment, there were novel findings for the individual assessments. Our finding that a minimum of three consultations need to be observed to provide feedback and generate learning in formative assessment has not previously been reported. With RCA new findings included the importance of the assessor rather than the learner selecting the records, and the assessor being mindful of the risk to the assessment when the selected records are of poor quality allowing the learner to embellish their recall of the consultation. The quality of RCA is known to be dependent on the memory and recall of the participant [14] and we found problems when records were not of recent consultations.

Miller and Archer completed a systematic review of workplace-based assessments and found there were few studies of the use of multiple assessments [10]. The only report of RCA in multiple assessments was combined with an OSCE examination [25]. RCA was similarly found to be a feasible, useful and acceptable addition to the existing assessment.

Concerns have been raised with many methods available for workplace-based formative assessment. Patient feedback may not reliably detect poor performers [26]. Multi-source feedback without facilitated feedback from a credible assessor may lack educational impact [27, 28]. Self-assessment of safety using a checklist [29] and the use of critical incident reports [30] have been proposed but are little studied.

Both the RCA and DO components of the assessment were considered to be useful in driving learning for registrars of all abilities. A formative approach was generally taken by the educators and registrars in the study. Although the ARCADO assessment tool required a summative assessment of overall competence for stage of training, the larger component of the tool promoted feedback across a wide range of knowledge, skills and attitudes. The design of the tool and the involvement of trained and experienced medical educators is likely to have helped avoid the well-known potential for formative assessment to be perceived as summative [31]. When this happens, learning is hindered by the reluctance of learners to expose weaknesses in a high-stakes setting.

Although workplace-based assessments are desirable settings to assess what a learner actually does, creating valid and reliable assessments in this setting can be difficult. In the workplace environment it is difficult to control the number of patients seen, patient mix and complexity, and the impact of team-based care on the assessment [32]. Our findings raise additional contextual concerns for RCA if the records are not selected randomly or where poor records provide an opportunity for embellishment by the registrar.

We primarily investigated ARCADO as a formative or low-stakes assessment. We found that ARCADO was useful in generating learning needs but medical educator participants were less confident in its use as an assessment of competence, particularly in lower performing registrars. Were ARCADO to be used in medium or high-stakes assessment, further evaluation and modification should be undertaken to improve validity and reliability, as well as consideration of how it sits with other assessments in a programmatic blueprint. In a high-stakes assessment, the *validity* could potentially be increased by stipulating that a certain number of reviewed charts relate to particular domains or subject areas. This would be similar to the previously recommended selection of specific patients for consultation observation in the ECTV [33]. However, there is a risk identified by our participants that a less random selection may inadvertently shift the assessment back from an assessment of ‘does’ to ‘shows how’, thus negating one of the advantages of adding RCA.

In a high-stakes assessment context, the number of observed and reviewed cases required for a *reliable* ARCADO assessment would need to be determined. While as few as ten charts [34] and ten observed consultations [13] have been noted to be required to achieve acceptable reliability, the number of each in a combined assessment is likely to be less.

The ARCADO assessment used was a modification of an existing method and was only trialled in one region. It was used by visiting GP medical educators rather than in-practice supervisors who may be less skilled as assessors. Registrars in remediation were excluded. Our results may not be transferable to other circumstances. This study did not examine whether the formative assessment resulted in change in learner behaviour or patient outcomes.

## Conclusion

We have found that ARCADO is a well-accepted workplace-based formative assessment that is capable of driving learning. It is perceived by learners and assessors to be a valid assessment that is flexible and time-efficient, and provides complementary insights into the consultation. Whilst there are some contextual factors to be considered in the implementation of ARCADO, it would appear to have utility as formative assessment and, subject to further evaluation, higher-stakes assessment.

## Abbreviations

ARCADO: adding random case analysis to direct observation
DO: direct observation of a consultation
ECTV: external clinical teaching visit
GP: general practice
OSCE: objective structured clinical examination
RCA: random case analysis
RTP: regional training provider
